# Understanding COVID-19-related myocarditis: pathophysiology, diagnosis, and treatment strategies

**DOI:** 10.1097/CP9.0000000000000046

**Published:** 2023-04-14

**Authors:** Hongyang Shu, Chunxia Zhao, Dao Wen Wang

**Affiliations:** 1 Division of Cardiology, Department of Internal Medicine, Tongji Hospital, Tongji Medical College, Huazhong University of Science and Technology; Hubei Key Laboratory of Genetics and Molecular Mechanisms of Cardiological Disorders, Wuhan 430030, China.

**Keywords:** COVID-19, Myocarditis, Physiopathology, Diagnosis, Therapeutics

## Abstract

Coronavirus disease 2019 (COVID-19) disease has infected nearly 600 million people, resulting in > 6 million deaths, with many of them dying from cardiovascular diseases. Severe acute respiratory syndrome coronavirus 2 (SARS-CoV-2) infection is caused by a combination of the virus surface spike protein and the human angiotensin-converting enzyme 2 (ACE2) receptor. In addition to being highly expressed in the lungs, ACE2 is widely distributed in the heart, mainly in myocardial cells and pericytes. Like other types of viruses, SARS-CoV-2 can cause myocarditis after infecting the myocardial tissue, which is attributed to the direct damage of the virus and uncontrolled inflammatory reactions. Patients with chest tightness, palpitation, abnormal electrocardiogram, and cardiac troponin elevation, should be suspected of myocarditis within 1–3 weeks of COVID-19 infection. When the hemodynamics change rapidly, fulminant myocarditis should be suspected. Cardiac ultrasound, myocardial biopsy, cytokine detection, cardiac magnetic resonance imaging, 18F-fluorodeoxyglucose positron emission tomography, and other examination methods can assist in the diagnosis. Although scientists and clinicians have made concerted efforts to seek treatment and prevention measures, there are no clear recommendations for the treatment of COVID-19-related myocarditis. For most cases of common myocarditis, general symptomatic and supportive treatments are used. For COVID-19-related fulminant myocarditis, it is emphasized to achieve “early identification, early diagnosis, early prediction, and early treatment” based on the “life support-based comprehensive treatment regimen.” Mechanical circulatory support therapy can rest the heart, which is a cure for symptoms, and immune regulation therapy can control the inflammatory storms which is a cure for the disease. Furthermore, complications of COVID-19-related myocarditis, such as arrhythmia, thrombosis, and infection, should be actively treated. Herein, we summarized the incidence rate, manifestations, and diagnosis of COVID-19-related myocarditis and discussed in detail the treatment of COVID-19-related myocarditis, especially the treatment strategy of fulminant myocarditis.

## INTRODUCTION

For 3 years, beginning from January 8, 2019, China has taken strict epidemic prevention and control measures following the outbreak of the coronavirus disease 2019 (COVID-19) pandemic in 2019. By 2023, China has downgraded the disease management from Class A to Class B in accordance with the country’s law on the prevention and treatment of infectious diseases. The COVID-19 strain of the Omicron virus quickly swept across the country, exerting tremendous pressure on the country’s medical system. Many people were admitted to the hospital due to chest tightness and palpitations and diagnosed with “COVID-19-related myocarditis.” There have been reports on social media that COVID-19 survivors die of myocarditis due to exercise in the short term, triggering a new round of panic. Therefore, it is necessary to provide a summary of the epidemiology, pathogenesis, diagnosis, and treatment of COVID-19-related myocarditis to improve the understanding of the disease.

## COVID-19 INFECTS MYOCARDIAL TISSUE

Severe acute respiratory syndrome coronavirus 2 (SARS-CoV-2) belongs to coronavirus, a large, enveloped single-stranded RNA virus found in humans and other mammals (such as dogs, cats, chickens, cattle, pigs, and birds). The diameter of SARS-CoV-2 ranges from 60 to 140 nm, with a sharp peak of 9 to 12 nm, which makes the virus particles exhibit the characteristics of the solar corona^[1]^. The viral particles contain four structural proteins: spike (S), envelope (E), membrane (M), and nucleocapsid (N). The virus genome is a single-stranded positive-strand RNA with a length of approximately 29.9 kb. The open reading frame in the genome was arranged in sequence as 5′-replicase (ORF1a/ORF1b) -S-ORF3a-ORF3b-E-M-ORF6-ORF7aORF7b-ORF8-N-ORF9a-ORF9b-ORF10-3′. Nucleocapsin N wraps viral RNA to form the core structure of the virus particle nucleocapsid, which is then wrapped by a double lipid membrane embedded with the S, M, and N proteins of COVID-19^[2]^.

After invading the human respiratory tract, the SARS-CoV-2 virus mainly depends on the receptor-binding domain (RBD) on the surface of the S protein to recognize the host cell receptor angiotensin-converting enzyme 2 (ACE2) and bind with it to infect the host cell^[3]^. The gene of SARS-CoV-2 frequently mutates during its prevalence and transmission in the population. When different subtypes or offspring of SARS-CoV-2 infect humans, they recombine and produce recombinant viral strains^[4]^. Some mutations or recombination will change the biological characteristics of the virus, for example, the mutation of specific amino acids on the S protein will lead to the enhancement of affinity between COVID-19 and ACE2 and the enhancement of replication and transmission in cells^[5]^. Some amino acid mutations in the S protein will increase the immune escape ability of the vaccine and reduce the cross-protection ability between different sub-branch mutants, leading to breakthrough infection and a certain proportion of reinfection^[6]^.

By the end of 2022, the World Health Organization had put forward five “variants of concern” (VOC)^[7]^, including Alpha (B.1.1.7)^[8]^, Beta (B.1.351)^[9]^, Gamma (P.1)^[10]^, Delta (B.1.617.2)^[11]^ and Omicron (B.1.1.529)^[12]^. The Omicron variant appeared in the population in November 2021. Compared with other VOC variants, such as Delta, its transmissibility and immune escape ability were significantly enhanced. It rapidly replaced the Delta variant as the global dominant epidemic strain in early 2022^[13]^. To date, five subtypes of omicron (BA.1, BA.2, BA.3, BA.4, BA.5) evolved into more than 750 sub-branches over a series of generations.

Evidence at home and abroad shows that the lung pathogenicity of the Omicron mutant strain is weakened, its clinical manifestation has evolved from pneumonia to upper respiratory tract infection, and most patients have mild symptoms^[14,15]^. However, among adults affected by COVID-19 Omicron, the cardiovascular system is still involved in a large proportion^[16]^. The incidence of acute myocardial injury in hospitalized patients ranges from 7% to 36%^[17]^, and the overall incidence of arrhythmia is 16.8%^[18]^. Furthermore, acute myocardial infarction^[19]^, heart failure^[20]^, and cardiogenic shock (CS) are often encountered.

In theory, the cells in cardiac tissue (including cardiac myocytes, fibroblasts, endothelial cells, and pericytes) are almost threatened by SARS-CoV-2 because of the expression of ACE2 or the influence of adjacent cells. Evidence of the damage of SARS-CoV-2 to myocardial cells was obtained from the autopsy of COVID-19 patients. SARS-CoV-2 nucleic acids were detected in 47 (48%) of 97 autopsies^[21]^. During the autopsy, myofibril fragmentation and nuclear destruction can be observed. Hypertrophic, deformed, and necrotic cardiomyocytes contain coronavirus particles, and degenerated cardiomyocytes are adjacent to lymphocytes^[22]^. Pericytes are a type of perivascular cell located near the lumen surface of blood vessels and are closely associated with endothelial cells. They are mainly distributed in the capillaries and the small veins behind the capillaries^[23]^. The analysis of a single-cell atlas of adult hearts showed that the expression of ACE2 was the highest in pericytes^[24]^. In the hearts of COVID-19 patients, a significant reduction in pericytes and pericyte apoptosis has been observed^[25]^. Primary human cardiac pericytes exposed to SARS-CoV-2 wild-type strain or α and δ variants can cause rare infection events, and exposure to recombinant S protein alone can cause changes in signal transduction and function of peripheral cells^[26]^. Fibroblasts are the most common type of cells in the heart. Although fibroblasts do not express or only express a small number of ACE2 in healthy hearts, they seem to lack the target of COVID-19 attack, however, after short-term exposure to SARS-CoV-2 protein, they still show obvious inflammation^[27]^. Furthermore, single-cell atlas analysis of autopsy tissue samples showed that the cell differentiation process in fibroblasts was significantly upregulated, suggesting that fibroblasts may also be affected by COVID-19 infection^[28]^. ENCODE data from the endothelium of arteries, veins, and microvascular beds showed that there was almost no basic ACE2 expression in the endothelial cells^[29]^, however, patients with COVID-19 had obvious endothelial dysfunction, including extensive thrombosis and endocarditis, which might be secondary to infection of adjacent cells or inflammatory damage^[30]^.

The researchers evaluated the replication ability of the SARS-CoV-2 original strain, Delta, and Omicron BA.1, BA.2, and BA.5 and their damage to myocardial cells. The results showed that BA.5 variants had stronger replication ability, more infectivity, stronger cell damage effect than BA.1. They were similar to Delta, however, the Delta variant had much stronger replication ability and destructive power in myocardial cells than BA.1 variant. The replication ability of BA.2 and BA.5 variants and their injury effect in cardiac myocytes are much stronger than those of BA.1, which is similar to the delta variant^[31]^.

## EPIDEMIOLOGY OF COVID-19-RELATED MYOCARDITIS

Since the 21st century, three major coronavirus outbreaks have been reported: SARS-CoV, Middle East respiratory syndrome coronavirus (MERS-CoV), and SARS-CoV-2, which are still prevalent worldwide. In 2002, the SARS-CoV pandemic broke out in Guangdong Province, China. There were 8,098 infected people worldwide, 774 of whom died, resulting in a 9.6% case fatality rate^[32]^. The outbreak of Middle East Respiratory Syndrome in the Arabian Peninsula in 2012 infected 2,562 people, with a mortality rate of approximately 34.4%^[33]^. The SARS-CoV-2 pandemic has infected more than 500 million people, with a global mortality rate of approximately 4%. Acute myocardial injury and myocarditis are as high as 62% of inpatients with severe SARS-CoV-2, and severe myocardial injury is closely related to high mortality^[34,35]^.

Myocarditis is a localized or diffuse inflammatory disease of the myocardium^[36]^. According to the severity of the patient’s condition, myocarditis can be divided into acute myocarditis and fulminant myocarditis. The onset of fulminant myocarditis is rapid, and the patient develops hemodynamic abnormalities and severe arrhythmia, which may be accompanied by respiratory failure and liver and kidney failure^[37]^.

There are several causes of myocarditis. The most common cause is viral infections, such as coxsackie B virus, echovirus, and poliovirus^[38]^. During the COVID-19 pandemic, COVID-19-related myocarditis is also common. According to data from the Centers for Disease Control and Prevention of the United States, 10–20 people out of every 100,000 people were diagnosed with myocarditis yearly before the COVID-19 epidemic^[38]^. Since March 2020, there have been approximately 146 patients with myocarditis per 100,000 people, much higher than the prevalence of COVID-19. A study of more than 56,000 hospitalized patients infected with COVID-19 in 23 hospitals in Europe and the United States reported that 2.4 of every 1000 people were definitely or probably suffering from acute myocarditis. Among them, 21 (38.9%) showed symptoms of fulminant myocarditis and required temporary mechanical circulatory support. The incidence rate of myocarditis in COVID-19 patients at autopsy is much higher than that in clinical assessment, which ranges from 2% to 7%^[39]^. A recently published study on patients with COVID-19 with elevated troponin showed that the incidence rate of myocarditis was only 7%, which may be related to the fact that the study only included patients with elevated troponin and survived during hospitalization, but did not include patients who may have died from myocarditis^[40]^.

## PATHOGENESIS OF COVID-19-RELATED MYOCARDITIS

**Figure 1.**
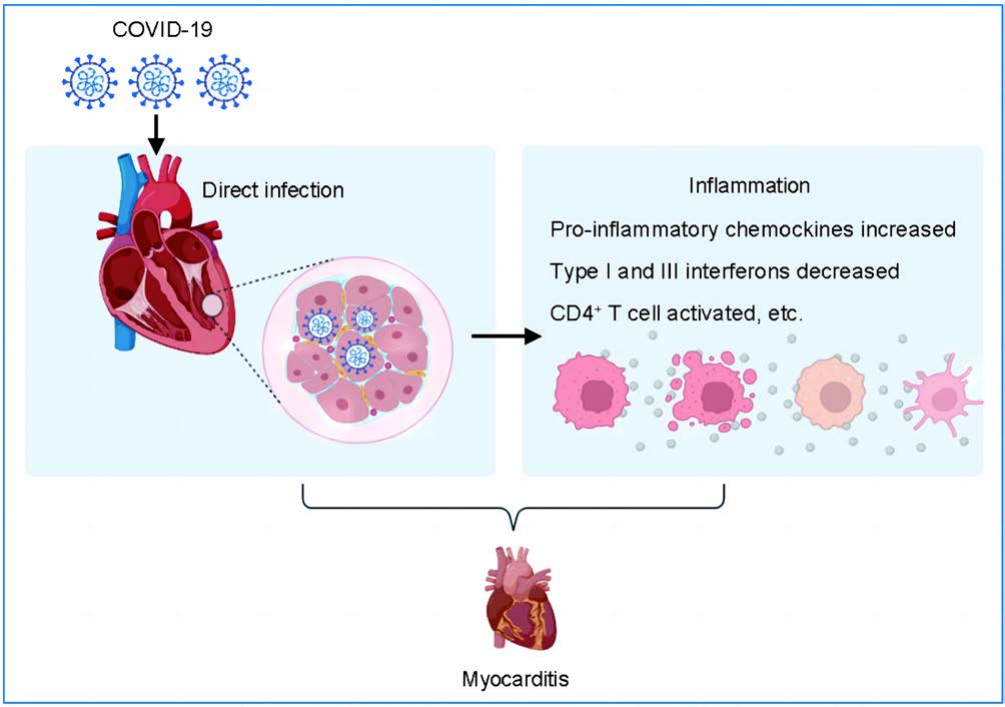
**Pathogenesis of COVID-19-related myocarditis**. COVID-19: coronavirus disease 2019; CD: cluster of differentiation.

The damage caused by SARS-CoV-2 infection can be divided into two stages: viral infection and uncontrolled inflammation (Figure 1). The pathogenesis of COVID-19-associated myocarditis is believed to be primarily related to uncontrolled inflammatory reactions^[41]^.

The viral infection stage is characterized by viral replication, resulting in direct virus-mediated tissue damage (Figure 1). After SARS-CoV-2 combines with ACE2 on the surface of the target cell membrane, the type 2 transmembrane serine protease (TMPRSS2) existing in the target cell promotes the uptake of the virus by splitting ACE2 and activating the SARS-CoV-2S protein. After the virus enters the cell, it releases its RNA, replicates, releases it, and further infects adjacent cells, causing tissue damage^[42,43]^. An autopsy revealed that the hypertrophic, deformed, and necrotic cardiomyocytes contain COVID-19 virus particles^[44]^. Furthermore, direct damage of the virus to the vascular system changes the vascular endothelium. It induces the activation of platelets, monocytes, and macrophages, thus promoting the release of tissue factor, von Willebrand factor, and factor VIII, resulting in thrombin and fibrin clot formation^[45]^, leading to myocardial tissue damage.

The second stage is a local inflammation and cytokine storm caused by tissue damage, that is, an uncontrolled systemic inflammatory reaction related to the release of a large number of pro-inflammatory cytokines [including interleukin-6 (IL-6), IL-1, IL-2, IL-10, tumor necrosis factor alpha (TNF-α), and interferon-γ] and coagulation dysfunction^[46–49]^. Excessive inflammatory reactions may be related to changes in the myeloid reaction and the production of pathogenic autoantibodies. The single-cell map of the immune response of patients with severe COVID-19 showed that during life-threatening COVID-19, the phenotype of peripheral immune cells was reconstructed^[50]^, including the downregulation of human leukocyte antigen class II and the emergence of a large number of immature neutrophils. Additionally, autoantibodies have been identified in severe COVID-19, including a high proportion of antibodies against nuclear antigens, phospholipids, T-cell antigens, B-cell antigens, chemokines, and cytokines^[51]^ (Figure 1).

An excessive inflammatory reaction is the core pathogenesis of COVID-19-associated myocarditis. Patients with myocarditis have higher inflammatory biomarkers, including increased ferritin, C-reactive protein, and cytokines (IL-2, IL-10, IL-5, etc.) than patients without myocarditis^[52,53]^. In cases of cardiac infection, myocardial tissue containing viral particles can be observed, however, the number is small. The median density of SARS-CoV-2 cells in the myocardium is 1 cell/cm^2^, and SARS-CoV-2 cannot be detected in some patients with myocarditis. In an endocardial biopsy, the density of cardiac CD68 macrophages and CD3 lymphocytes is relatively high^[54]^, and their density is positively correlated with the duration of symptoms of cardiac injury. Myocardial biopsy samples of SARS-CoV-2-related fulminant myocarditis showed interstitial inflammatory infiltration mainly composed of macrophages and T cells. CS is a critical manifestation of myocarditis. The SARS-CoV-2 genome and significant immunoreactivity of viral nucleocapsid proteins cannot be detected in the myocardial tissue of patients with CS^[55]^. The histopathology of multiple system inflammatory syndrome in children caused by SARS-CoV-2 shows significant inflammatory infiltration in the myocardium. However, SARS-CoV-2 nucleic acids cannot be detected^[56]^. Studies on animal models and cell-based analysis after SARS-CoV-2 infection, as well as serum and transcriptional profiles of patients with COVID-19, showed that the levels of type I and III interferons decreased while the expression of chemokines and IL-6 increased. High levels of pro-inflammatory cytokines and chemokines, such as MCP-1, IL-6, granulocyte colony-stimulating factor (G-CSF), macrophage inflammatory protein 1α (MIP-1α), monocyte chemotactic protein-3 (MCP-3), and interferon-γ inducible protein-10 (IP-10) in hospitalized patients with SARS-CoV-2 infection were positively correlated with disease severity in hospitalized patients with the disease. In addition, CD4 T cells of patients infected with SARS-CoV-2 are rapidly activated to produce large amounts of granulocyte-macrophage colony-stimulating factor (GM-CSF) and IL-6. GM-CSF further activates CD14^+^ and CD16^+^ inflammatory mononuclear cells to produce more IL-6, thus further aggravating damage to target organs. Approximately 10% of COVID-19 patients develop acute post-COVID-19 syndrome after rehabilitation, and about 25%–50% of them reported tachycardia or palpitation^[57]^, known as “post-COVID-19 tachycardia syndrome,” which has been confirmed to be related to excessive inflammatory reaction^[58]^.

## MANIFESTATION AND DIAGNOSIS OF COVID-19-RELATED MYOCARDITIS

**Figure 2.**
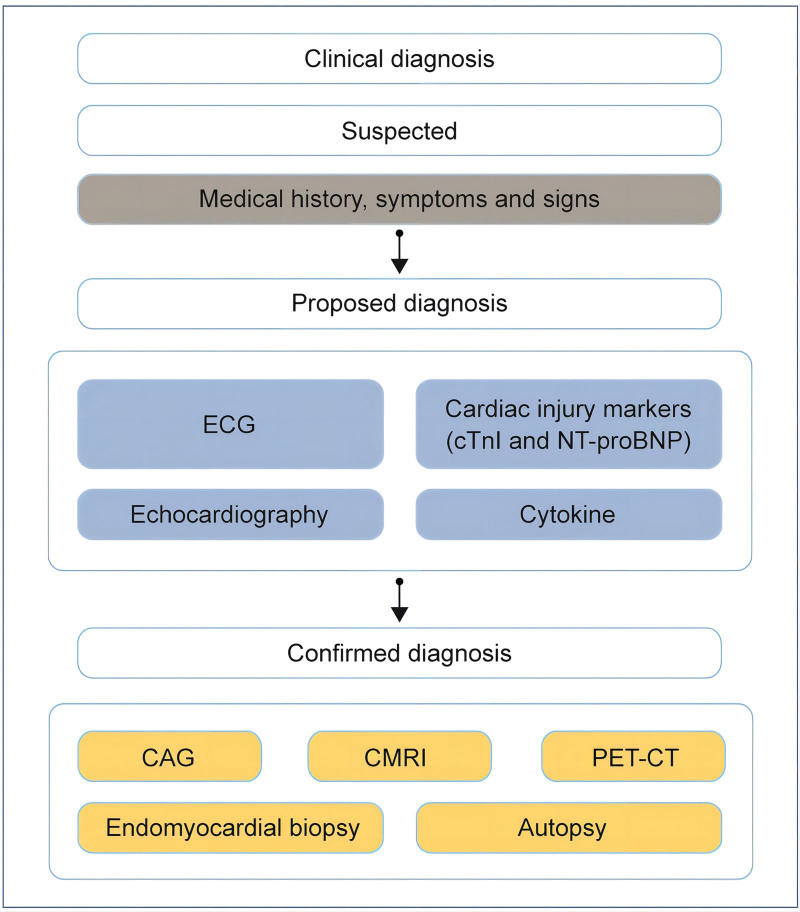
**Flow chart of myocarditis diagnosis**. ECG, echocardiography, cTnI, NT-proBNP, CAG, and blood cytokine detection need to be completed immediately. Selecting an appropriate time for CMRI and endomyocardial biopsy according to the situation. CAG: coronary angiography; CMRI: cardiac magnetic resonance imaging; cTnI: cardiac troponin; ECG: electrocardiogram; NT-proBNP: N-terminal pro b-type natriuretic peptide; PET-CT: positron emission tomography/computed tomography.

The manifestations and diagnosis of COVID-19-related myocarditis are similar to myocarditis caused by other viruses^[41]^. After COVID-19, most patients experience fever, fatigue, muscle soreness, nausea, vomiting, and other symptoms. After 1-3 weeks, they have heart symptoms, such as fatigue, palpitation, chest tightness, shortness of breath, and even cardiac failure symptoms, such as dyspnea. When patients have cardiac manifestations, especially palpitation, and shortness of breath, within 3 weeks or at the remission stage after COVID-19 infection, they should be aware of the possibility of COVID-19-related myocarditis (Figure 2). During physical examination, if the patient’s first heart sound at the apex of the heart is significantly weakened, there is diastolic galloping rhythm, pericardial friction sound, congestive heart failure, or shock. the serum cardiac troponin (cTnI) or high-sensitivity troponin is significantly increased, and electrocardiogram shows new arrhythmias, including atrioventricular block, sinus block, multi-source paired ventricular premature beats, atrial flutter or fibrillation, and more than two leads show horizontal or oblique downward movement or abnormal Q wave, COVID-19-related fulminant myocarditis should be suspected. However, most patients with COVID-19-related myocarditis are not severe and are easy to ignore. Patients have palpitations, shortness of breath, and elevated cTnI I levels. If the patient has a sudden onset of the severe hemodynamic disorder (including hypotension or shock) and arrhythmia (malignant arrhythmias such as ventricular tachycardia and ventricular fibrillation), COVID-19-related fulminant myocarditis is suspected (Figure 2). When the patient’s echocardiography showed the following characteristics: diffuse reduction of ventricular wall motion, a significant decrease in left ventricular ejection fraction, and decrease in left ventricular long-axis strain, laboratory tests showed that the myocardium was severely damaged, and the level of inflammatory factors was significantly increased in severe myocarditis. In most patients with myocarditis, speckle tracking echocardiography can clearly observed obvious abnormality^[59]^, COVID-19 patients have extensive left ventricular systolic dysfunction, which is related to the severity of the disease^[60]^, and acute myocardial infarction, stress cardiomyopathy, and septic cardiomyopathy should be excluded. In particular, coronary angiography should be performed for some patients, and patients can be clinically diagnosed with COVID-19-related fulminant myocarditis (Figure 2). Furthermore, there are a few cases of fulminant myocarditis with SARS-CoV-2 only invading the cardiac conduction system, which are mainly manifested as malignant arrhythmias, such as high atrioventricular block, frequent ventricular tachycardia and ventricular fibrillation, etc. Highly sensitive-cTnI/cTnI and B-type natriuretic peptide (BNP)/N-terminal prohormone of BNP (NT-proBNP) are not significantly increased, just because of edema by a cytokine storm. These patients should undergo inflammatory factor detection, echocardiography, and myocardial biopsy; if necessary, cardiac magnetic resonance imaging (CMRI) or 18F-fluorodeoxyglucose positron emission tomography, should be performed as soon as possible for diagnosis^[61]^.

## TREATMENT OF COVID-19-RELATED MYOCARDITIS

**Figure 3.**
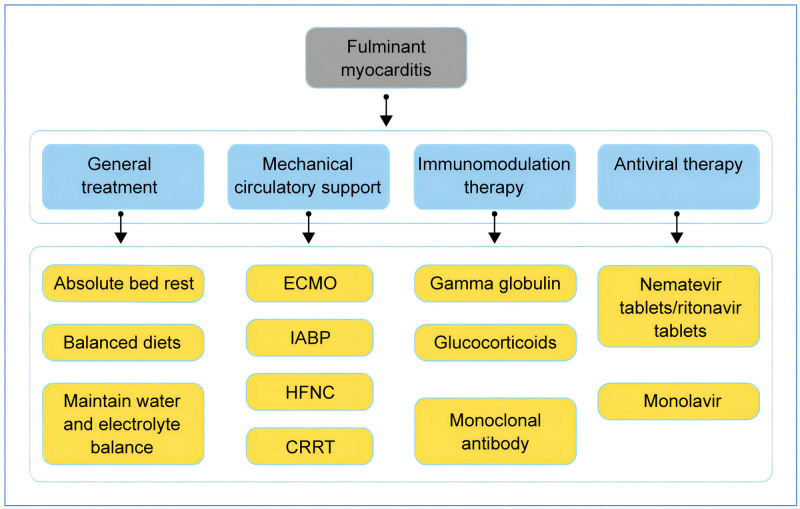
**Treatment flow chart of patients with myocarditis in the acute phase**. CRRT: continuous renal replacement therapy; ECMO: extracorporeal membrane oxygenation; HFNC: high-flow nasal cannula; IABP: intra-aortic balloon pump.

SARS-CoV-2 has led to multisystem damage. Scientists and clinicians have made concerted efforts to seek treatment and preventive measures. Currently, many efforts have been made to develop new drugs for SARS-CoV-2 and to test previous drugs^[62]^, including antiviral drugs (such as nucleotide analogue)^[63]^, antibodies (such as convalescent plasma and high immunoglobulin)^[64]^, anti-inflammatory drugs (dexamethasone and statins)^[65]^, targeted immunomodulatory therapy (such as tocilizumab, sarilumab, anakinra, and ruxolitinib), anticoagulants (such as heparin)^[66]^, and anti-fibrosis drugs (such as tyrosine kinase inhibitors)^[67]^. However, treatment of SARS-CoV-2 infection is still based on symptomatic treatment and subsequent supportive care. The main interventions included bed rest, adequate heat, water balance, oxygen therapy, antiviral therapy, and supportive treatment. In addition, some patients experience combined bacterial and fungal infections and need broad-spectrum antibiotic treatment (Figure 3).

Gamma globulin is generally believed to be effective against SARS-CoV-2 infection, but the administration of immunosuppressants such as corticosteroids is very cautious^[68]^. The Chinese guidelines recommend the use of corticosteroids in acute respiratory distress syndrome (ARDS) derived from COVID-19 in the short and medium term at low to medium doses. However, the Centers for Disease Control and Prevention (CDC) and the World Health Organization do not recommend the administration of these drugs because the administration of endogenous or exogenous glucocorticoids will inhibit the immune response, hinder virus clearance, and lead to the aggravation of infectious diseases^[69]^. In treating children’s multisystem inflammatory syndrome caused by COVID-19, it has been confirmed that the use of moderate doses of glucocorticoids can significantly inhibit inflammatory responses, regulate immunity, and effectively improve the end-organ dysfunction and shock status of patients^[70,71]^. In reported cases of SARS-CoV-2-related fulminant myocarditis, mechanical circulation support therapy and immunoglobulin combined with steroid shock therapy also showed good therapeutic effects^[72]^. In addition, some immune-specific drugs have been proven to be beneficial, especially those against the IL-6 pathway, including tocilizumab and sarilumab^[73]^. Janus kinase-signal transducer and activator of transcription protein (JAK-STAT) signaling pathway inhibitors such as axitinib and tofacitinib show potential^[74]^.

Hypertension and diabetes are risk factors for cardiovascular diseases and are susceptible to COVID-19 infection. Among COVID-19 inpatients, hypertension is the most common complication, followed by diabetes and cerebrovascular disease. In addition, COVID-19 patients with hypertension, diabetes, and other risk factors are more likely to progress to critical patients^[75]^. Therefore, for patients with cardiovascular risk factors in COVID-19, the above risk factors should be actively controlled. At present, it has been confirmed that angiotensin-converting enzyme inhibitors (ACEI), a first-line antihypertensive drug that can theoretically increase the expression of ACE2, not only does not increase the risk of infection of COVID-19 but also reduces the mortality of COVID-19 and the risk of rehospitalization^[76]^.

In addition, COVID-19 causes many complications, including venous thromboembolism^[77]^. The overall incidence rate of COVID-19-related venous thromboembolism was 17%, that of deep venous thrombosis was 12.1%, and that of pulmonary embolism was 7.1%^[78]^. The use of anticoagulants has been proven to reduce stroke, myocardial infarction, and all-cause mortality compared with the use of anticoagulants^[79]^.

However, there are currently no clear recommendations for treating COVID-19-related myocarditis. For COVID-19-related fulminant myocarditis, achieving “early identification, early diagnosis, early prediction, and early treatment” is emphasized based on the “life support-based comprehensive treatment regimen”^[80]^.

General treatment should be actively performed for common acute myocarditis. The main contents include (1) bed rest to avoid emotional excitement and fluctuation; (2) ensuring sufficient nutrition intake and electrolyte balance, and maintaining internal environment stability; (3) monitoring blood pressure, heart rate, electrocardiogram, myocardial enzymology, and other indicators; (4) giving standardized and effective oxygen therapy measures according to the condition, including nasal catheter, mask oxygen, and nasal high-flow oxygen therapy; (5) antiviral treatment with nimatvir tablets/ritonavir tablets, azudine tablets, and monovir capsules; (6) use of immunomodulatory therapy represented by glucocorticoids and gamma globulin; (7) drugs improving the myocardial metabolism of patients like trimetazidine; and (8) application of β-receptor blockers and ACEI.

The major problems in patients with fulminant myocarditis are acute circulatory failure and CS, which should be treated in accordance with the “life support-based comprehensive treatment regimen.” The core contents of treatment include (1) mechanical life support: mechanical circulatory devices are used to maintain circulation stability and ensure organ perfusion so as to rest the failed heart, rather than cardiotonic and vasoactive drugs to raise blood pressure, if necessary, ventilator-assisted breathing, temporary pacemaker implantation, and blood purification treatment should be carried out; (2) immunoregulation therapy: sufficient doses of glucocorticoid and immunoglobulin are used to regulate immunity; and (3) use of neuraminidase inhibitors. Oral or nasal administration of oseltamivir (75 mg twice daily) helps reduce myocardial injury. In addition, the antiviral drugs targeting COVID-19 can block virus replication and reduce viral load, which can help reduce the virus’s direct and indirect immune damage to the myocardium (Figure 3).

### Mechanical circulatory support

Intra-aortic balloon pump (IABP) counterpulsation: Multi-center research and experience reports have confirmed that early application of IABP in patients with fulminant myocarditis can significantly increase systolic blood pressure by more than 20 mmHg, reduce the increased sinus heart rate by approximately 10 beats per minute, reduce the dosage of vasoactive drugs, and improve short-term and long-term prognosis. If IABP is administered early, more than 70% of patients can maintain circulation stability without adding other mechanical circulatory devices, such as Extracorporeal membrane oxygenation (ECMO). Therefore, IABP should be used preferentially when patients with fulminant myocarditis have early symptoms of shock, such as hypotension and rapid heart rate.

ECMO: Venoarterial-ECMO (VA-ECMO) is supported by a large amount of clinical data on the treatment of fulminant myocarditis. Venovenous ECMO (VV-ECMO) or venoarteriovenous ECMO (VAV-ECMO) can be considered in patients with respiratory failure. In short, when IABP is still insufficient to correct circulatory disorders, it is recommended. When the patient has serious circulatory disturbance, serious left ventricular insufficiency or cardiac arrest, and cardiopulmonary resuscitation, ECMO should be started immediately, and an IABP should be implanted simultaneously.

Impella left ventricular assist device or artificial heart: In addition to IABP and ECMO, Impella and Tandem Heart are used. The Impella can maintain blood flow, which allows sufficient cardiac output to ensure organ perfusion. It also reduces left ventricular afterload and allows the left ventricle to rest. Successful treatment of treating COVID-19-related fulminant myocarditis with Impella alone^[72]^.

### Other mechanical life support treatment

Non-invasive mechanical ventilation: It is recommended for patients with hypoxemia/respiratory failure complicated by COVID-19-related myocarditis who have difficulty breathing or respiratory rate >20 breaths/min, can fully cooperate with and adapt to ventilator ventilation and is expected to have short-term remission.

Invasive mechanical ventilation: patients with COVID-19-related fulminant myocarditis who cannot adapt to non-invasive mechanical ventilation, cardiopulmonary resuscitation, or lack of non-invasive ventilation support should receive invasive mechanical ventilation as soon as possible.

Transnasal high-flow oxygen therapy (HFNC): For patients with hypoxemia/acute type I respiratory failure, HFNC should be used instead of traditional oxygen therapy (COT) to reduce the tracheal intubation rate and the need for respiratory support upgrading.

Continuous renal replacement therapy (CRRT): CRRT can continuously remove toxins and inflammatory factors, reduce the damage caused by inflammatory storms, regulate the body fluid and acid-base balance, and stabilize the internal environment. CRRT should be used early in patients with COVID-19-related fulminant myocarditis complicated by renal dysfunction or acute left heart failure.

### Immunomodulation therapy

In fulminant myocarditis, excessive immune activation and inflammatory storms can lead to severe myocardial damage. Because of this pathophysiological basis, “immunomodulation” treatment, including the adequate doses of glucocorticoid and sufficient dose of immunoglobulin, should be taken, rather than “immunosuppression” treatment using cytotoxic drugs. If necessary, monoclonal antibodies of cytokines should be tried, which can block the pathogenesis, reduce inflammatory edema and anti-shock, relieve clinical symptoms, save patients’ lives, and improve prognosis.

Early administration of adequate doses of glucocorticoids: patients with COVID-19-related fulminant myocarditis should be started on glucocorticoids immediately after diagnosis. Specifically, methylprednisolone 200–500 mg (or 3–8 mg/kg) is administered intravenously every day (in the case of an emergency, methylprednisolone can be administered based on intravenous dexamethasone 10-20 mg). After 3 to 5 consecutive days, the dose was gradually decreased according to the condition (usually starting from the left ventricular EF value greater than 40%). Before discharge, intravenous methylprednisolone was changed to oral prednisone (20–40 mg/day) for 1–3 months. During the follow-up period, cardiac function, cTnI level, inflammatory factor level, CMRI or myocardial biopsy showing the degree of myocarditis and edema, drug tolerance, drug withdrawal, and adjustment treatment should be considered according to the patient’s symptoms.

Monoclonal antibody to IL-6: Tocilizumab is a recombinant humanized IL-6 receptor monoclonal antibody of the immunoglobulin G1, which imitates the natural antibody produced by the immune system. It can be used in critically ill patients with significantly elevated IL-6 levels.

### Antiviral therapy

Nematevir tablets/ritonavir tablets, is administered to adult patients with mild and moderate disease and high-risk factors for progression to severe disease within 5 days.

Azudine tablets is used to treat adult patients with moderate COVID-19 infection.

Monolavir capsules is administered to adult patients with mild and moderate disease and high-risk factors for progression to severe disease within 5 days.

Monoclonal antibody, ambavir monoclonal antibody/romisvir monoclonal antibody injection, is administered to treat adults and adolescents (12 to 17 years old, weight ≥ 40 kg) who are light, medium, and have high-risk factors for progression to severe disease.

## TREATMENT OF COMPLICATIONS OF COVID-19-RELATED MYOCARDITIS

### Prevention and treatment of arrhythmia

About one fifth of patients with fulminant myocarditis have arrhythmias. Measures to prevent arrhythmia include (1) intravenous dexamethasone 10–20 mg immediately after diagnosis; (2) the minimum dose of dopamine and m-hydroxylamine/or norepinephrine used to maintain the MAP at 60–65 mmHg (not too high); and (3) a person with shortness of breath should receive mechanical ventilation. The treatment of arrhythmia should follow existing arrhythmia guidelines.

### Prevention and treatment of thrombus

Patients with fulminant myocarditis are prone to severe coagulation dysfunction and thrombosis due to shock, inflammatory storm, and other damage to the endothelium. Measures to prevent thrombus include (1) correcting CS and containing inflammatory storm; (2) monitoring the blood coagulation function and finding the DIC precursor in time; (3) correcting repeated cardiac arrest, circulatory instability, and sympathetic system excitation; (4) avoiding the administration of norepinephrine, m-hydroxylamine, pituitrin, and other antihypertensive drugs in large doses for a long time; and (5) immediate infusion of fresh plasma, cryoprecipitates, and platelets.

### Prevention and treatment of infection

During the treatment of fulminant myocarditis, because of the use of large doses of glucocorticoids, it is recommended to use broad-spectrum antibiotics to prevent infection, actively look for infection factors, and use sufficient antibiotics as early as possible according to the pathogen culture and drug sensitivity test results. During the anti-infection process, a continuous dynamic evaluation should be performed to adjust the treatment strategy at any time.

## Conclusions

Compared with previous coronavirus outbreaks, such as SARS-CoV in 2002 and MERS-CoV in 2012, SARS-CoV-2 has a lower mortality rate, however, its transmission rate is higher. The global pandemic caused by SARS-CoV-2 has led to thousands of deaths worldwide. The best treatment strategy for COVID-19 remains unknown. The involvement of the cardiovascular system is an essential reason for COVID-19 to cause critical illness. For the treatment of myocarditis associated with COVID-19, we should rely on the “life support-based comprehensive treatment regimen” to carry out mechanical circulation support, immune regulation and antiviral treatment, emphasizing the principle of “early identification, early diagnosis, early prediction, and early treatment,” which can significantly reduce mortality and save the lives of a large number of patients.

## FUNDING

This work was supported by grants from the National Natural Science Foundation of China (82241034 and C-0052).

## AUTHOR CONTRIBUTIONS

DWW and CXZ participated in research design. HYS participated in the writing of the paper. DWW revised the final manuscript.

## ACKNOWLEDGEMENTS

We thank the language editing service provided by editage (https://www.editage.cn/).

## CONFLICT OF INTEREST STATEMENT

The authors declare that they have no conflict of interest with regard to the content of this manuscript.

## DATA SHARING STATEMENT

All data generated or analyzed during this study are included in this published article.
